# Supplementary figures and images for: Differential allelic representation (DAR) identifies candidate eQTLs and improves transcriptome analysis
Source: PLoS Comput Biol. 2024 Feb 12;20(2):e1011868. doi: 10.1371/journal.pcbi.1011868 (PMC10890730; doi:10.1371/journal.pcbi.1011868)

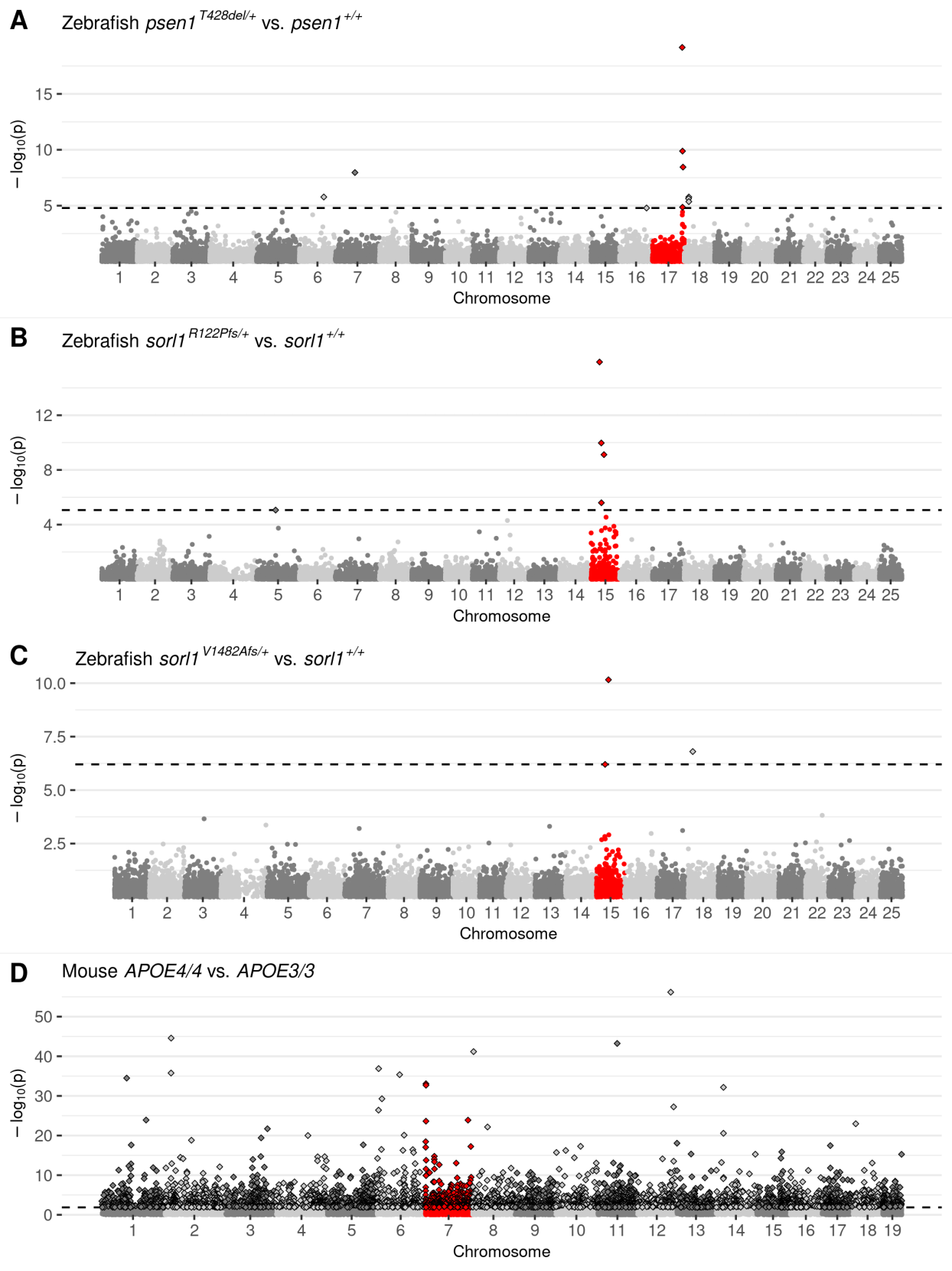

Supplement: S1 Fig — A) zebrafish EOfAD-like psen1T428del/+ vs. psen1+/+ B) zebrafish EOfAD-like sorl1 R122Pfs/+ vs. sorl1+/+ C) zebrafish EOfAD-like sorl1V1482Afs/+ vs. sorl1+/+ D) mouse APOE4/4 vs. APOE3/3. Non-random accumulation of DEGs was tested using a Bonferroni-adjusted Fisher’s exact test p-value for enrichment of DE genes on the mutant chromosome, A) psen1T428del/+ vs. psen1+/+: p = 8.63e-3, B) sorl1 R122Pfs/+ vs. sorl1+/+: p = 1.92e-4, C) sorl1V1482Afs/+ vs. sorl1+/+: p = 9.24e-2, D) APOE4/4 vs. APOE3/3: p = 1.00. Genes are plotted along the x-axis based on their chromosomal position in alternating shades of grey for visual distinction between chromosomes. Genes on the chromosome containing the mutation are highlighted in red. The raw p-values are plotted along the y-axis at the -log10 scale such that the most significant genes exist at the top of the plot. The cut-off for gene differential expression (FDR-adjusted p-value < 0.05) is indicated by a dashed horizontal line. Genes classified as differentially expressed under this criterion are represented as diamonds with a black outline. (TIFF) [file pcbi.1011868.s001.tiff]

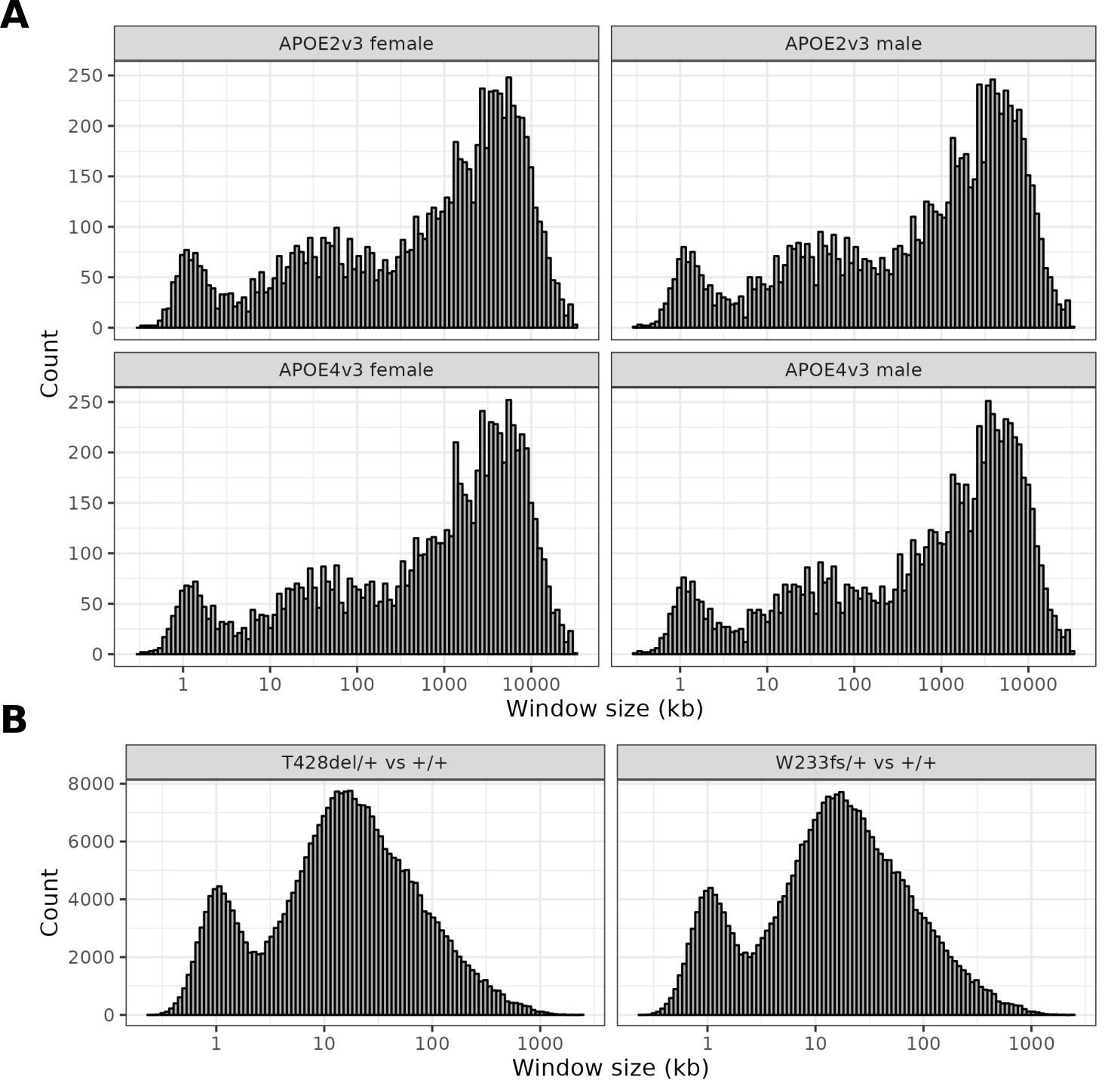

Supplement: S2 Fig — Window sizes are determined based on the genomic distance between 11 subsequent SNPs. Zebrafish datasets have a greater number of variant sites distributed across the genome relative to mouse due to the lack of isogenicity, resulting in much smaller window sizes. (TIFF) [file pcbi.1011868.s002.tiff]

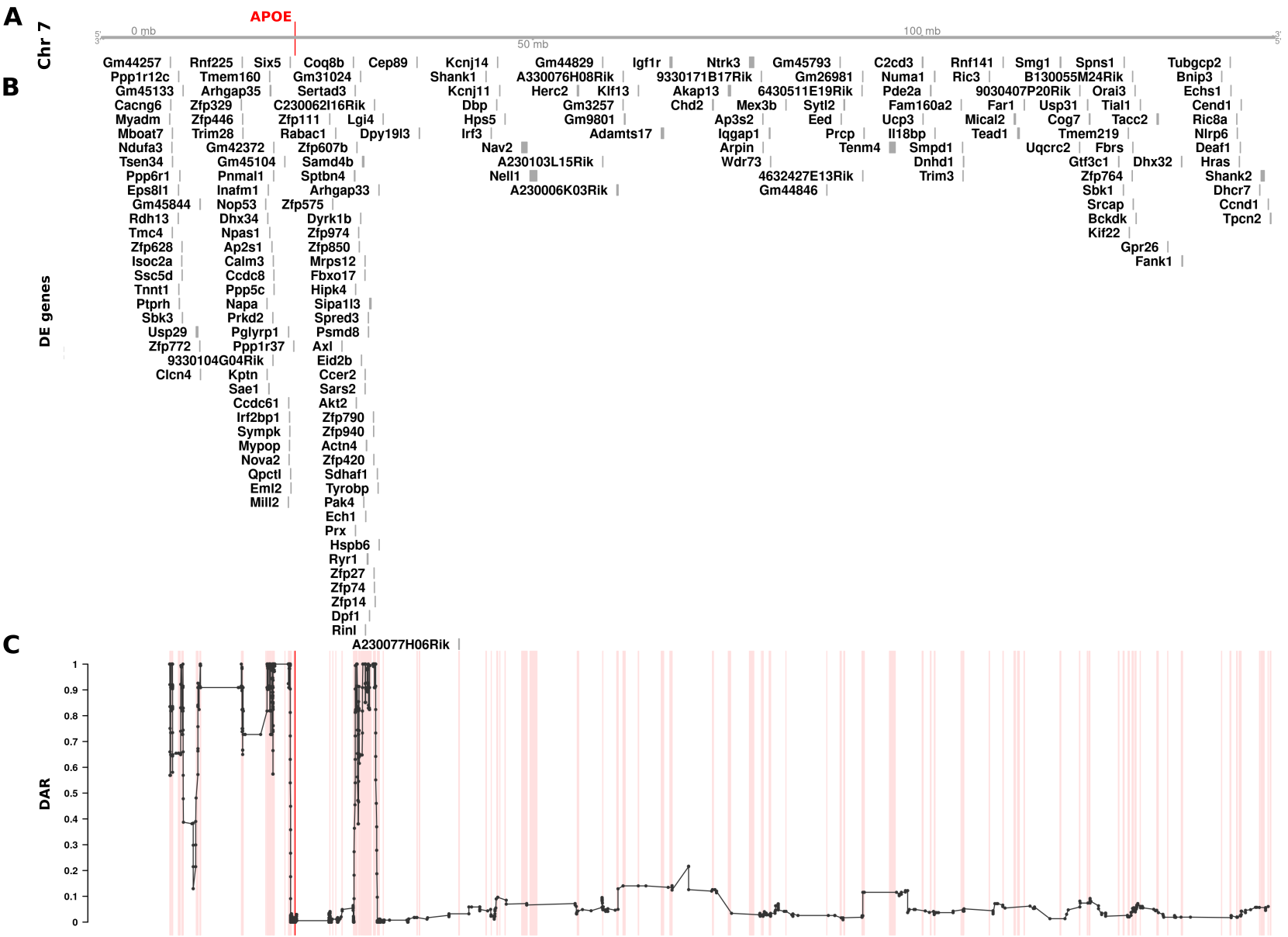

Supplement: S3 Fig — The plot contains four sets of information represented by separate tracks horizontally. Track A represents the axis of Chromosome 7. The position of the APOE gene is marked and labelled in bold red. Track B displays differentially expressed genes according to their positions along the chromosome. 183 of 1126 total genes (16.25%) on Chromosome 7 that were expressed in the dataset were classified as DE (FDR < 0.05). Track C shows the trend in DAR as a connected scatterplot with each point in black representing the DAR value at a single nucleotide variant position (elastic sliding window size = 11 variants). Positions of the DE genes shown in track B are indicated by light red lines. (TIFF) [file pcbi.1011868.s003.tiff]

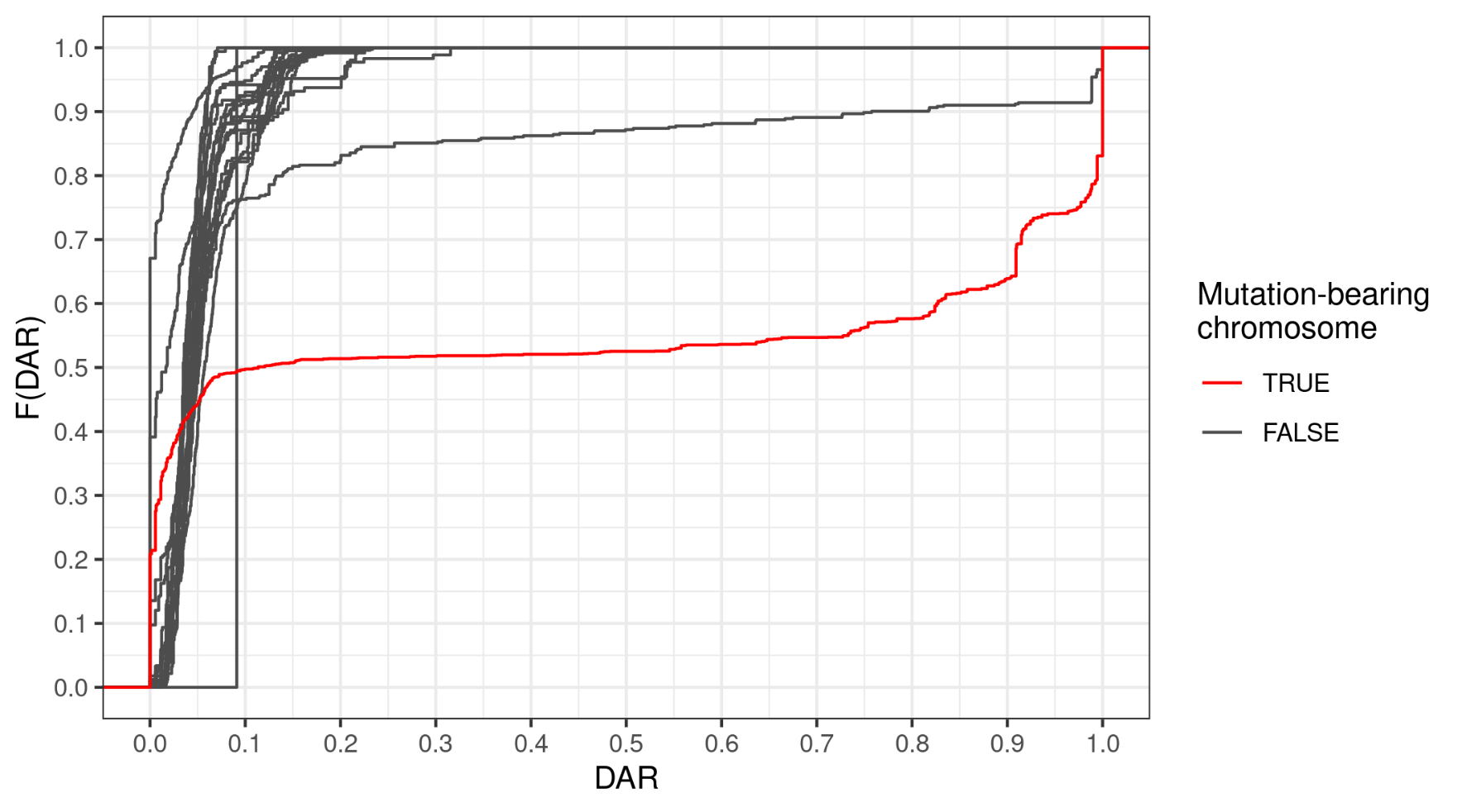

Supplement: S4 Fig — The mutant chromosome exhibits the most regions of high DAR. (TIFF) [file pcbi.1011868.s004.tiff]

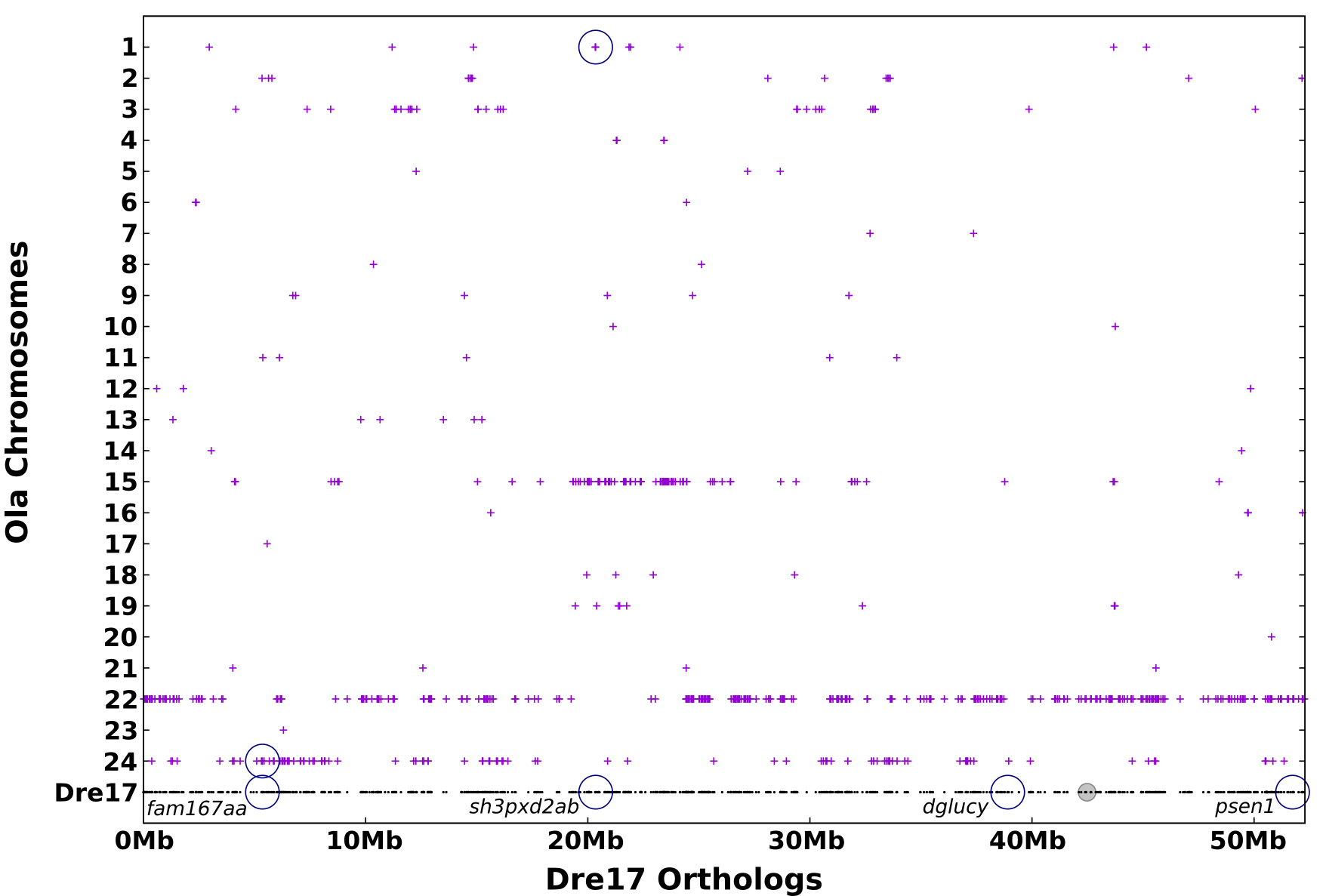

Supplement: S5 Fig — Genes are plotted along the x-axis based on their position on Chromosome 17 in zebrafish, while the y-axis indicates the chromosome they are located on in medaka. The four DE genes predicted to have orthologues are circled in blue. The two genes without a secondary blue circle plotted along the y-axis (dglucy and psen1), were predicted by the Synteny Database to exist on alternate scaffolds of medaka Ensembl version 71, which are not plotted. The smaller grey-filled circle indicates the centromere of Dre Chromosome 17. (TIFF) [file pcbi.1011868.s005.tiff]

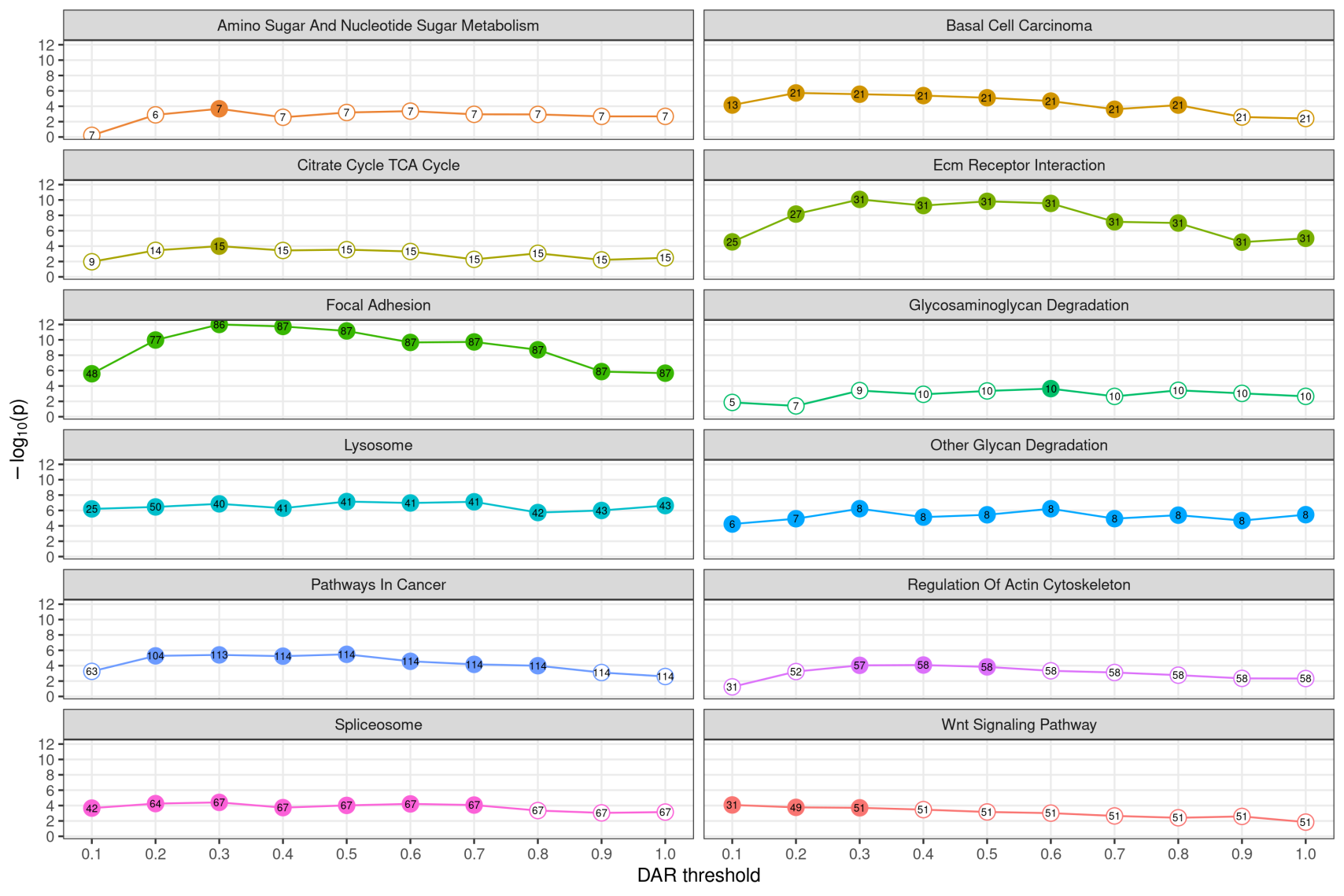

Supplement: S6 Fig — The gene sets displayed are those that achieved significance for at least one DAR threshold value. p-values are plotted on a -log10 scale along the y-axis such that the most significant results exist at the top of each graph. Each position along the x-axis represents a different DAR gene exclusion threshold. Dots on the graph filled with colour indicate that the gene set was classified as significantly enriched (FDR-adjusted p-value < 0.05). The number inside a dot corresponds to the number of leading-edge genes that contributed to the respective gene set’s enrichment score. (TIFF) [file pcbi.1011868.s006.tiff]

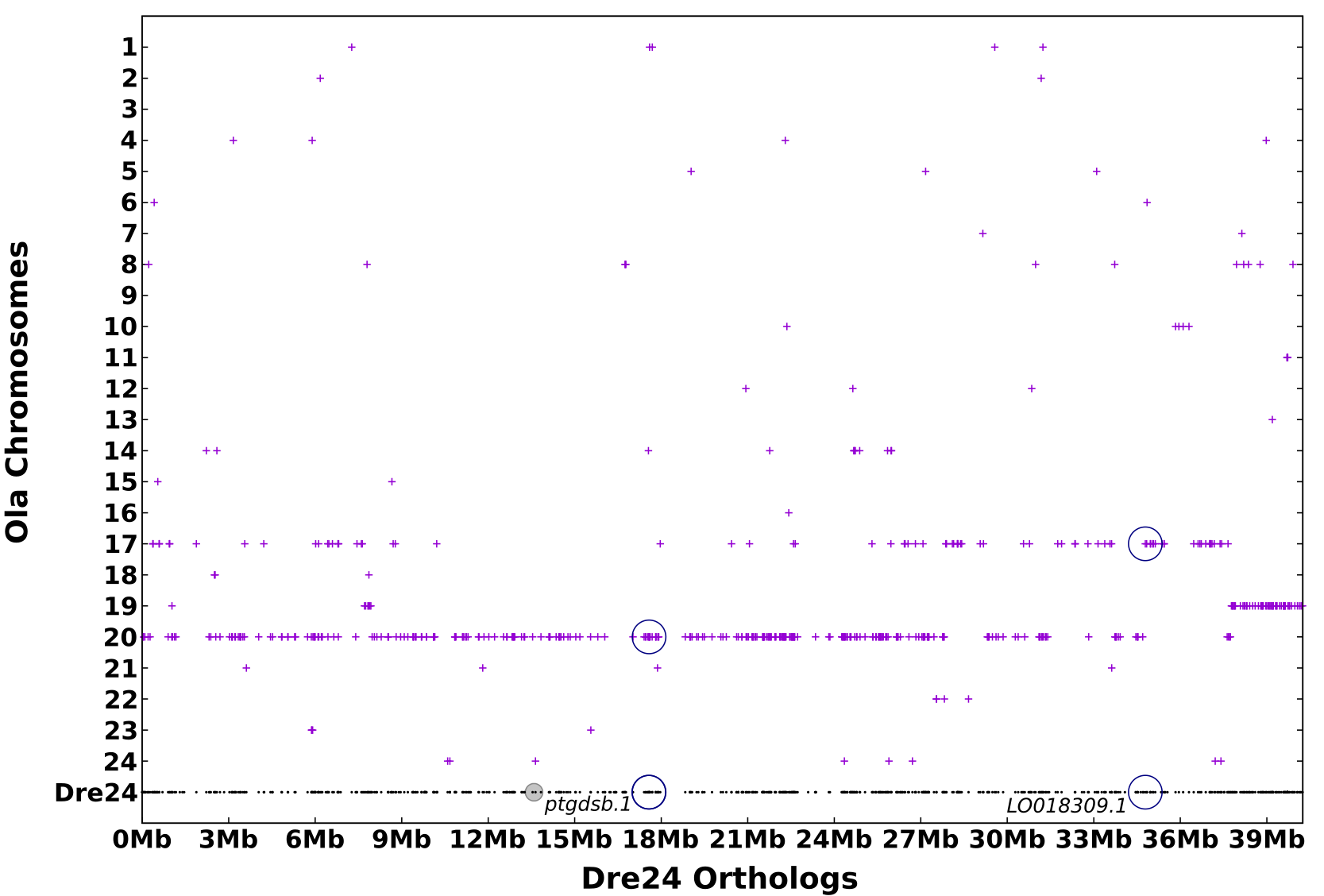

Supplement: S7 Fig — Genes are plotted along the x-axis based on their position on Chromosome 24 in zebrafish, while the y-axis indicates the chromosome they are located on in medaka. The two DE genes predicted to have orthologues are circled in blue. The smaller grey-filled circle indicates the centromere of Dre Chromosome 24. (TIFF) [file pcbi.1011868.s007.tiff]

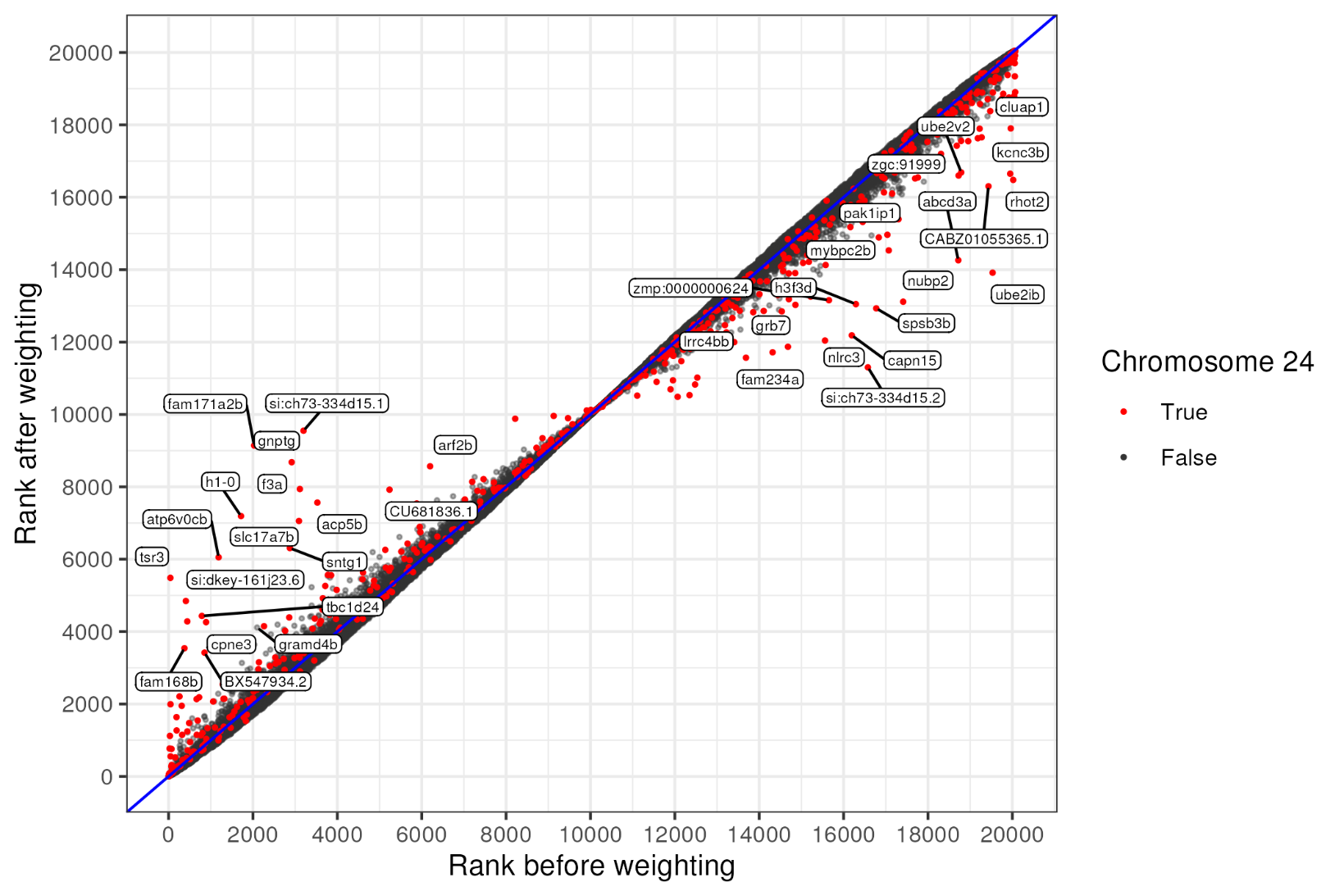

Supplement: S8 Fig — Genes that are plotted close to the blue diagonal line are least impacted by the weighting method. Genes that exist on the mutant chromosome (Chromosome 24) are coloured red, while those that exist on other chromosomes are coloured black. Genes that were most substantially affected (rank change > 2000) are labelled with their respective gene symbol. (TIFF) [file pcbi.1011868.s008.tiff]

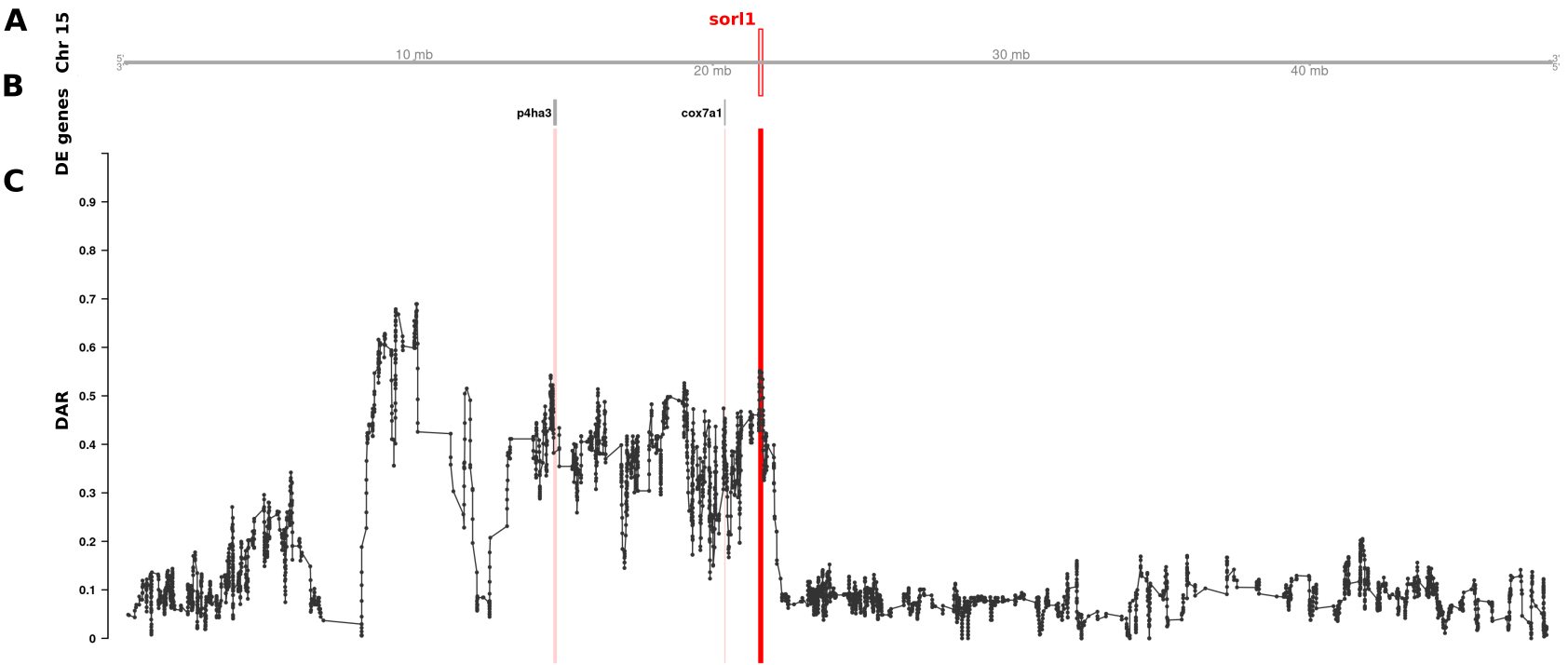

Supplement: S9 Fig — The plot contains four sets of information represented by separate tracks. Track A represents the axis of Chromosome 15. The position of the sorl1 gene is marked and labelled in bold red. Track B displays differentially expressed genes according to their positions along the chromosome. Track C shows the trend in DAR as a connected scatterplot with each point representing the DAR value at a single nucleotide variant position (elastic sliding window, n = 11 variants). Positions of the DE genes shown in track B are indicated by light red lines. (TIFF) [file pcbi.1011868.s009.tiff]

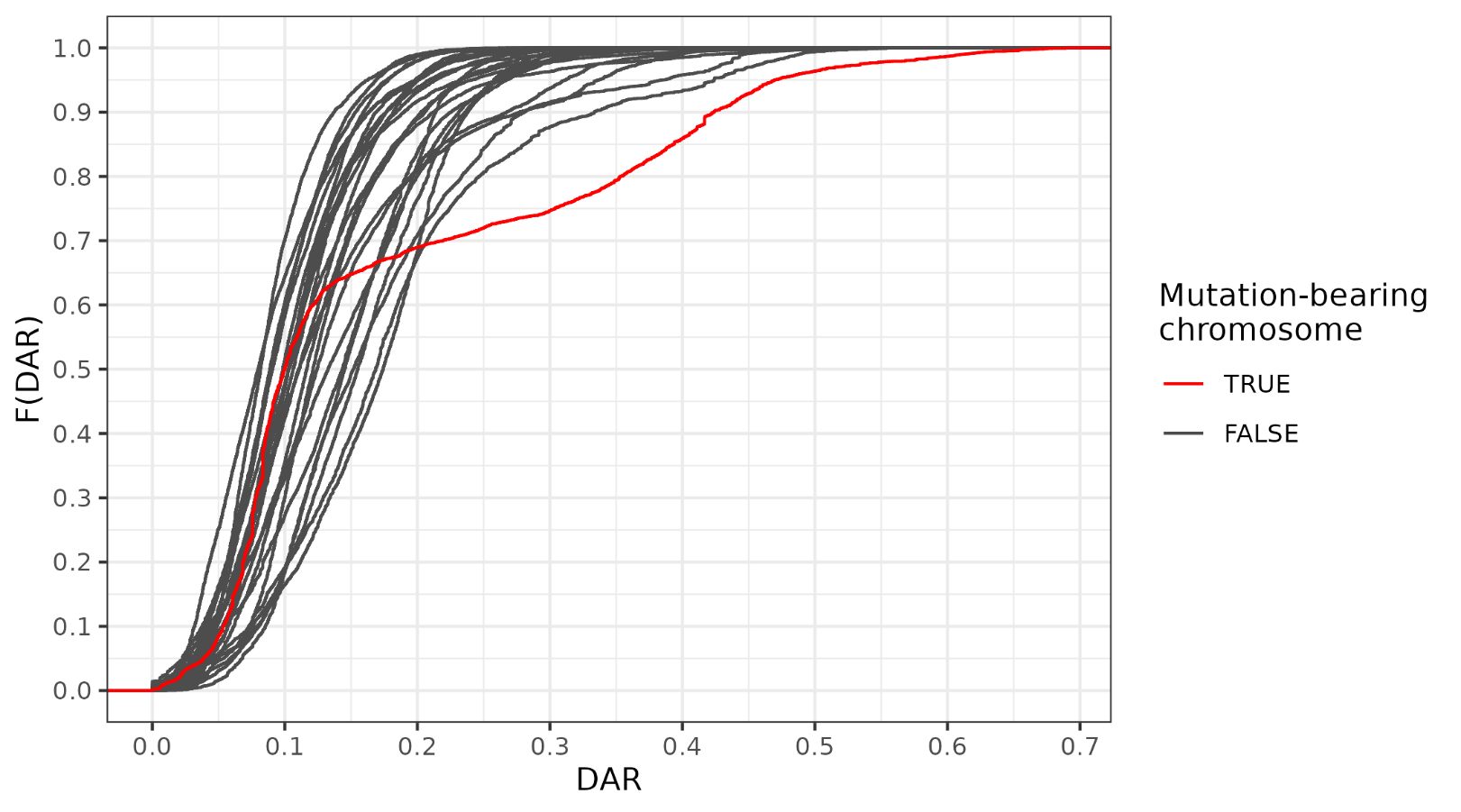

Supplement: S10 Fig — The mutant chromosome exhibits the greatest number of regions of high DAR. (TIFF) [file pcbi.1011868.s010.tiff]

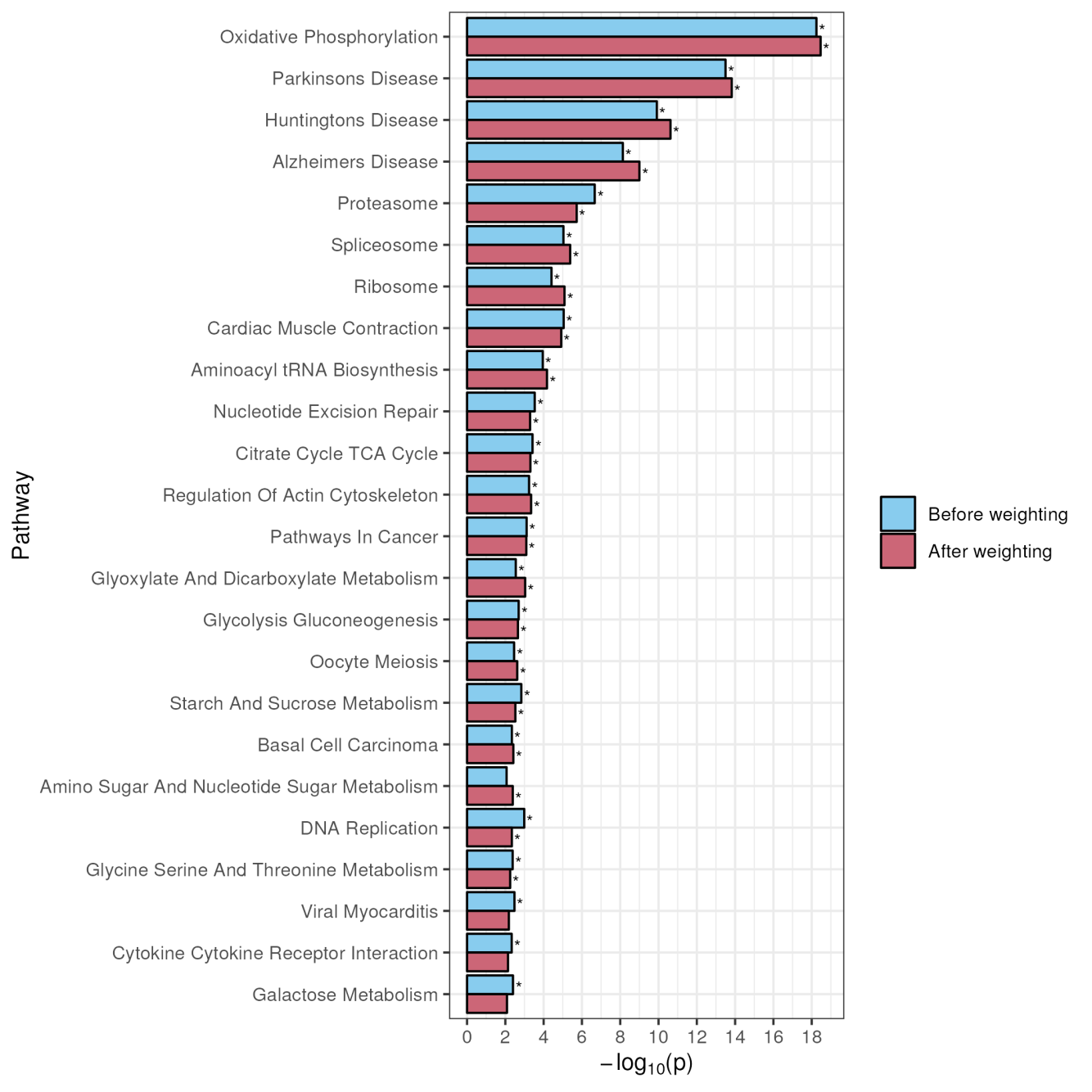

Supplement: S11 Fig — An asterisk is used to denote that the pathway was determined to be significantly enriched. (TIFF) [file pcbi.1011868.s011.tiff]

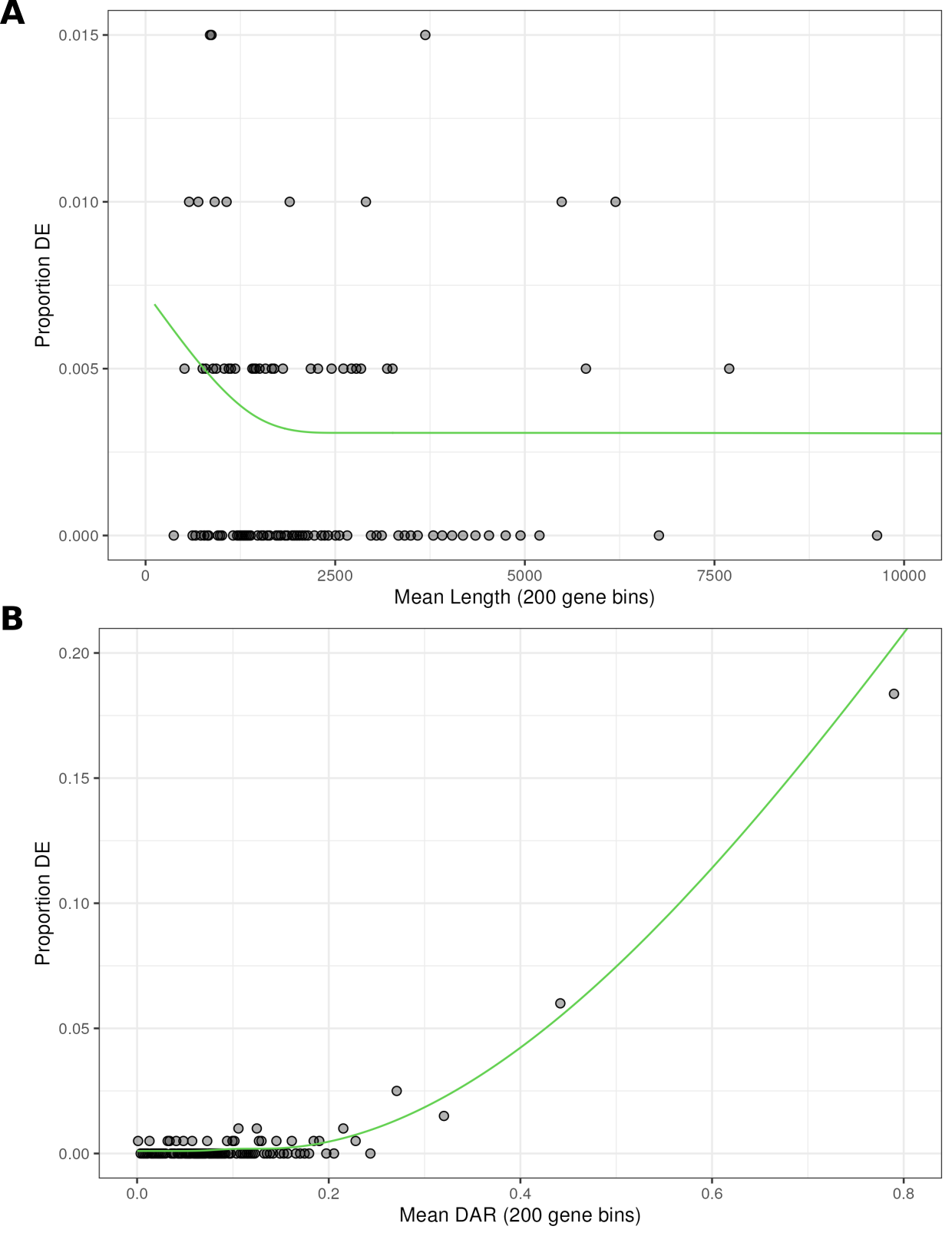

Supplement: S12 Fig — The resulting fit of the Probability Weighting Function (PWF) on the nagluA603Efs dataset for bias data A) Median transcript length B) DAR. The points indicate the proportion of DE genes for bias data in 200 gene bins. The green line represents the monotonic spline fitted when calculating the PWF. Minimal transcript length bias is observed relative to DAR bias. (TIFF) [file pcbi.1011868.s012.tiff]
