# Supplementary material for: Differential allelic representation (DAR) identifies candidate eQTLs and improves transcriptome analysis
Source: PLoS Comput Biol. 2024 Feb 12;20(2):e1011868. doi: 10.1371/journal.pcbi.1011868 (PMC10890730; doi:10.1371/journal.pcbi.1011868)
Supplement: Attachment — Submitted filename: response_to_reviewers_final.docx [file pcbi.1011868.s013.docx]

Revision of PCOMPBIOL-D-23-00494 for re-submission including responses to reviewers’ comments

Dear *PLOS Computational Biology*,

Thank you for the reviews of our manuscript,

“Differential allelic representation (DAR) identifies candidate eQTLs and improves transcriptome analysis”

We have now modified the manuscript in response to the comments from the reviewers. Below, the points raised by the reviewers are shown in bold text and our responses are given beneath each point in plain text.

We have also attached a “Compare Documents” version of the manuscript showing the changes made in the revised version to aid in the examination of those changes.

We hope that the changes are sufficient to allow publication of the manuscript in *PLOS Computational Biology*.

Sincerely,

Michael Lardelli

Response to reviewers’ comments

Reviewer #1: This paper investigates the phenomenon of functionally related genes becoming linked over evolution. The authors analyse several different mutants in both mouse and zebrafish which show an excess of differentially expressed (DE) genes on the same chromosome as the mutation. They propose a statistic (DAR) to distinguish between genes that are truly DE as a result of the mutation and those that appear to be DE due to the segregation of linked eQTLs into the two different sample groups. They provide a SnakeMake workflow to process RNA-seq including calculating DAR. Using DAR, the authors show, in a comparison of two different human APOE alleles introduced into mice, that the linked DE genes are more likely to be caused by eQTLs. In contrast, two psen1 mutations in zebrafish, show low DAR values across the whole of the chromosome with the mutation. This suggests that eQTLs are likely not the cause of these DE genes. Further, the authors suggest that these genes have become linked over the course of evolution. They look at Medaka as an example. Four of the seven DE genes in the psen1(W233fs) comparison (including psen1 itself) are located on the same Medaka chromosome. Of the other three, two had identifiable Medaka orthologues that are located on different Medaka chromosomes. The authors suggest that these two genes (fam167aa and sh3pxd2ab) have been captured at some point since the divergence of Medaka and zebrafish.

To investigate the effect of distinguishing true DE genes from those caused by eQTLs, the authors look at the effect on the gene set enrichment methods, ROAST and GSEA. They do this in another zebrafish example (naglu-A603Efs) by using a thresholding approach to progressively remove genes with high DAR scores that are more likely to represent DE genes caused by eQTLs. ROAST, possibly due to its methodology seems to be quite robust to the inclusion of eQTL-driven DE genes, whereas terms enriched using GSEA fluctuate more as the threshold changes. The authors propose that for GSEA, rather than using the DAR score to remove genes from the gene list, it can be used to downweight suspect DE genes in the input. Using this DAR-weighting strategy, one of the gene sets expected to be enriched (Glycosaminoglycan Degradation) becomes significantly enriched when previously it wasn't.

More and more comparative transcriptomic datasets are being generated from samples not from inbred lines. Therefore, these issues are likely to become more widespread. The analysis is well done, and all the relevant code is available. The Rmarkdown produced html files make browsing the results really easy.

Major points:

It would be very helpful to have a table with the analysed experiments and number of total and mutation-linked DE genes in each.

Thank you for this suggestion. We agree that providing readers with a summary of the experiments and numbers of DEGs/CC-DEGs is useful. We have added this table to the beginning of the results section so that readers can familiarise themselves with the data presented in following sections. We have also included a column indicating the significance classification from testing for over-representation of CC-DEGs using Fisher’s Exact Test.

The analysed DE lists seem very short (apart from the APO mouse data), so it would be good to see a couple of examples where their method is applied to DE lists longer than 50. For example, White et al. has a few suitable RNA-seq experiments.

Unfortunately, we could not find a suitable dataset from the 9 RNA-seq experiments presented by White et al. involving mutant lines. All 9 of these datasets were generated from pools of individuals (embryos or larvae) which compromises our methods for DAR analysis. While the variant calling software (specifically GATK HaplotypeCaller) is capable of handling non-diploid samples in some cases, the high ploidies encountered in pooled experiments are known to cause performance issues. We have previously tested this in our *fmr1* dataset (mentioned in the Introduction) where samples consisted of pooled embryos, but analysis was hindered by high memory requirements and excessively long processing times. Additionally, DAR analysis was developed to work directly from genotype calls assuming samples correspond to individuals. Implementing our approach on this data would therefore require a substantial reconstruction of the variant calling workflow and DAR analysis methods, which would be inconsistent with the rest of the manuscript.

While the DE list is still somewhat small, the naglu^A603Efs^ comparison resulted in 53 DE genes, which serves as an example of DAR analysis in zebrafish on a longer DE gene list alongside the mouse APOE data. We do agree that it would be preferable to include more examples with greater numbers of DE genes. However, we do not believe that the additional work justifies the value this would add to the manuscript.

The authors look at two methods of Gene Set enrichment testing (ROAST and GSEA). Frequently use tools such as PANTHER, gProfiler, Ontologizer and topGO use different enrichment statistics. It would be really useful to see what effect DAR-based thresholding has on one of these methods as a comparison.

Thank you for the suggestion. We looked into the over-representation analysis tools listed above but decided to use a method that we are familiar with, *GOseq.* *GOseq* performs over-representation analysis while also accounting for selection bias in RNA-seq data, which violates the assumptions of a typical hypergeometric test. We looked for over-representation of DE genes in GO terms for the *naglu^A603Efs^* comparison at each threshold, using median transcript length as the bias data for each gene. This approach returned similar outcomes to that of ROAST, most likely because over-representation analysis is also an exclusive test. We decided not to include this in the manuscript, but the results can be found in the 211130_Q96K97del_A603fs/docs/more_enrichment.html document. During our exploration of *GOseq*, however, we noticed that the input bias data could represent any property of a gene that influences its classification as DE. As we believe DAR provides a good estimate of this, we compared the results of using transcript length or DAR as bias data for *GOseq* analysis. We found that using DAR increased the significance of the three significant GO terms that are physiologically relevant to the *naglu^A603Efs^* mutation. We prefer this approach over thresholding as it uses statistical weighting methods as opposed to the removal of data, and have added our findings to the end of the Results and Discussion sections.

I can't reconcile the data in the 211130_Q96K97del_A603fs repository (211130_Q96K97del_A603fs-master/docs/enrichment.html) and the -log10(p) values plotted in Figure 8. The text states that initially five pathways were significant, but the table shows 10. Am I looking at the wrong table?

Thank you for spotting this inconsistency in the rendered html document. This results from an oversight regarding reproducibility within the msigdbr() function, which was used to obtain gene sets for enrichment analysis. It appears the document was rendered after upgrading the msigdbr package to a newer version than was previously used to produce the results reported in the manuscript. This likely occurred when using rmarkdown::render_site(); our usual workflow to ensure all documents are up-to-date. The issues lies within the babelgene package (a dependency of msigdbr), which pulls the mappings of zebrafish orthologs to human genes, as required for the construction of gene sets. Unfortunately the msigdbr() function does not possess arguments to control this behaviour in a reproducible manner, and therefore the package version dictates the output of the function. We successfully reproduced the results reported in the manuscript by replicating the environment within a fresh docker container created from the bioconductor/bioconductor_docker:RELEASE_3_16 image. The html report with results that correspond to those reported in the manuscript has been re-rendered and committed to the GitHub repository. A note about reproducibility has been prefaced within the document, and session information is also reported to provide users with the information required to successfully reproduce the analysis. We appreciate your observation and will opt for alternative ways to obtain gene sets in our future analyses.

Also, for Supplementary Figure 10, the text states

"Three of the pathways (Viral myocarditis, Cytokine-Cytokine Receptor Interaction and Galactose metabolism) that were initially classified as significant, but were on the verge of non-significance, were shown to lose significance after DAR-weighting."

In the Figure, 'Cytokine-Cytokine Receptor Interaction' has an asterisk both before and after weighting and I can't locate 'Galactose metabolism' on the plot.

The text says that this shows the data for sorl1R122Pfs/+ vs wt, but the terms look like they represent the V1482Afs_het results (210216_sorl1_snv-master/docs/enrichment.html).

Thank you again for noticing these issues. The inconsistency between the description of GSEA results and their depiction in Supplementary Figure 10 is also a result of the analysis reproducibility issue described in the previous point. The same procedure has been performed on the 210216_sorl1_snv repository and Supplementary Figure 10 has been replaced with the original figure described by the text. The interpretation that the data actually represents the *sorl1^V1482Afs^*/+ vs wt results instead of *sorl1^R122Pfs^*/+ vs wt is also correct. The text has been adjusted accordingly. We have additionally noticed that the associated Supplementary Figures 8 and 9 show results from the *sorl1^R122Pfs^*/+ vs wt comparison, but were intended to show the *sorl1^V1482Afs^*/+ vs wt comparison. We have now corrected these figures and legends appropriately. We apologise for the mix-up when attaching these files.

Minor points:

It would be useful context to also state in the text for each comparison how many DE genes there were in total.

Thank you for the suggestion. With the addition of a summary table displaying the numbers of DEGs and CC-DEGs at the beginning of the Results section, we have chosen not to add the number of DEGs in the text. We hope that the table will act as a sufficient resource for this information that readers can refer to.

For the APOE2/3 comparison only the data from male mice is shown. Are the same CC-DEGS seen in the comparison in female samples?

We initially included only male data as the visualisation showed a similar trend between DAR and DE genes in both the male and female comparisons. However, we realise that the intersect size between male and female CC-DEGs is also interesting information that should be reported. We found that from 99 and 183 CC-DEGs that exist in the male and female samples respectively, the intersect size is 59 CC-DEGs. We have now added this into the results section, and added the respective figure for female samples as a supplementary figure. Thank you for this constructive feedback.

The distribution of window sizes is show for the mouse data. Are the sizes in zebrafish comparable?

The window sizes in zebrafish are much smaller due to the lack of isogenicity. Variant sites are reported when an allele differs from the reference genome in at least one sample. This results in a relatively larger number of variants distributed across chromosomes, and hence smaller window sizes. We have added an additional panel to Supplementary Figure 2 for comparison purposes. Thank you.

For the DAR weighting method, the ranking of genes that change the most is shown (Supplementary Figure 7). It would be interesting to also see the effect on the DE genes present on Chr 24.

We agree that distinguishing between genes that do/do not exist on Chromosome 24 adds useful information to the figure. We have updated Supplementary Figure 7 by highlighting points corresponding to Chromosome 24 genes and have plotted them on top of other genes to ensure their visibility.

In Supplementary Figure 4 and 6, what do the smaller grey-filled circle represent?

The smaller grey circle in Supplementary Figures 4 and 6 represents the centromere of the zebrafish reference chromosome. Thank you for noticing that we missed a description for this element of the plots. We have now adjusted the figure legends accordingly.

Reviewer #2: Baer et al. develops a method to analyze transcriptomic datasets in order to shed light on the effect of mutation. In particular, they highlight issues with the existing methods and proposes their method as an alternative. I must admit that the paper could have been written in a better way. Even the abstract was very difficult to understand. While the introduction was much better, some of the other sections were obscure. It would be great if the authors rewrite the various sections for clarity. The question the authors are asking is important. The analysis seems solid. The comparisons, exclusion of false eQTLs, along with the identification of CC-DEGs not necessarily eQTLs, are commendable. Please find my comments is below.

Thank you. We agree that this paper is quite technical in nature and that can sometimes make interpretation of some sections somewhat difficult. However, it was not made clear which aspects of narrative are most confusing and so, in rewriting the Results and Discussion sections, we risk creating a new version of the paper that is similarly difficult. The paper as written went through extensive revision before submission as it was passed around all of the co-authors. Also, Reviewer 1 appeared largely satisfied with the presentation style. Therefore, we have not opted for extensive rewriting although the Abstract has indeed been modified and, hopefully, this increases its clarity.

1) Gene expression is bursty for a large number of genes. While the mechanism for bursting is not fully understood, it can give rise to bimodality in gene expression. Without accounting for such variability in gene expression will lead to incorrect conclusions while carrying out inferences. It is important that the authors address this issue.

Thank you. From what we understand, transcriptional bursting causes variability in gene expression between cells of the same population. In bulk RNA-seq data, because gene expression is summarised across tissue, we expect this variability to have less impact on the resulting inferences. There are numerous sources of bias that are unexplored in RNA-seq data and the intention of this manuscript is to specifically examine the impact of unequal distributions of polymorphic loci between experimental sample groups, i.e. differential allelic representation. Transcriptional bursting is another unexplored mechanism that may contribute to bias in RNA-seq data (particularly single cell RNA-seq), and so we have now mentioned it in the beginning of the Discussion section.

2) In ‘Using a DAR metric to exclude probable eQTLs improves gene set enrichment analyses‘ section, it's remarkable that the Glycosaminoglycan Degradation pathway does not attain statistical significance at any DAR threshold higher than 0.6. This could mean something physiologically relevant about this pathway that the authors should explore more.

Thank you. It is difficult to ascertain any physiological relevance of *Glycosaminoglycan Degradation* not attaining statistical significance above a DAR threshold of 0.6. We believe that this pathway was indeed perturbed in our mutant samples. However, this was obscured by the relatively high significance of biased genes that existed in regions of DAR > 0.6. This can be attributed to the methodology of GSEA that uses a ranked list approach as described in the Discussion section. To make our reasoning clearer, we have modified paragraph 5 of the Discussion section to explicitly suggest why it is that *Glycosaminoglycan Degradation* only attains significance at a DAR threshold of 0.6. We hope that this satisfies your comment.

3) In the introduction, the authors write, “Positive selection for the pair of alleles can eventually drive them to fixation in the population – they become the only extant alleles for those two genes”. The ‘only extant alleles’ is not clear to me. What about the possibility of new mutations introducing genetic diversity to the population, resulting in emergence of new alleles? If a new allele pair arises that provides an even greater fitness advantage?

Thank you. We have now modified the third paragraph of the Introduction to clarify that we are talking about the new chromosomal arrangement becoming fixed in the population (rather than the particular alleles in linkage disequilibrium) and that new allelic variants can arise with time.

We would like to thank the Reviewers for their valuable comments that have, we believe, led us to significantly improve the manuscript.

Sincerely,

Michael Lardelli on behalf of the co-authors
